# Hyperinsulinism associated with *GLUD1* mutation: allosteric regulation and functional characterization of p.G446V glutamate dehydrogenase

**DOI:** 10.1186/s40246-020-00262-8

**Published:** 2020-03-06

**Authors:** Karolina Luczkowska, Caroline Stekelenburg, Frédérique Sloan-Béna, Emmanuelle Ranza, Giacomo Gastaldi, Valérie Schwitzgebel, Pierre Maechler

**Affiliations:** 1 Department of Cell Physiology and Metabolism, University of Geneva Medical Center, 1206 Geneva, Switzerland; 2 Faculty Diabetes Center, University of Geneva Medical Center, 1206 Geneva, Switzerland; 3 Pediatric Endocrine and Diabetes Unit, Department of Pediatrics Gynecology and Obstetrics, University Hospitals of Geneva, Geneva, Switzerland; 4 Department of Genetic Medicine and Development, Faculty of Medicine, University of Geneva, 1211 Geneva, Switzerland; 5 Department of Genetic Medicine and Laboratory, University Hospitals of Geneva, 1211 Geneva, Switzerland; 6 Division of Endocrinology, Diabetology, Hypertension and Nutrition, Geneva University Hospitals, 1211 Geneva, Switzerland

**Keywords:** Hyperinsulinism/hyperammonemia syndrome, *GLUD1*, Glutamate dehydrogenase, Allosteric regulation

## Abstract

**Background:**

Gain-of-function mutations in the *GLUD1* gene, encoding for glutamate dehydrogenase (GDH), result in the hyperinsulinism/hyperammonemia HI/HA syndrome. HI/HA patients present with harmful hypoglycemia secondary to protein-induced HI and elevated plasma ammonia levels. These symptoms may be accompanied by seizures and mental retardation. GDH is a mitochondrial enzyme that catalyzes the oxidative deamination of glutamate to α-ketoglutarate, under allosteric regulations mediated by its inhibitor GTP and its activator ADP. The present study investigated the functional properties of the GDH-G446V variant (alias c.1496G > T, p.(Gly499Val) (NM_005271.4)) in patient-derived lymphoblastoid cells.

**Results:**

The calculated energy barrier between the opened and closed state of the enzyme was 41% lower in GDH-G446V compared to wild-type GDH, pointing to altered allosteric regulation. Computational analysis indicated conformational changes of GDH-G446V in the antenna region that is crucial for allosteric regulators. Enzymatic activity measured in patient-derived lymphoblastoid cells showed impaired allosteric responses of GDH-G446V to both regulators GTP and ADP. In particular, as opposed to control lymphoblastoid cells, GDH-G446V cells were not responsive to GTP in the lower range of ADP concentrations. Assessment of the metabolic rate revealed higher mitochondrial respiration in response to GDH-dependent substrates in the GDH-G446V lymphoblastoid cells compared to control cells. This indicates a shift toward glutaminolysis for energy provision in cells carrying the GDH-G446V variant.

**Conclusions:**

Substitution of the small amino acid glycine for the hydrophobic branched-chain valine altered the allosteric sensitivity to both inhibitory action of GTP and activation by ADP, rendering cells metabolically responsive to glutamine.

## Introduction

The incidence of congenital hyperinsulinism is estimated at 1 in 50,000 (e.g., in the USA) to 1 in 2500 in certain populations (e.g., in Saudi Arabia) live births [1, 2]. Mutations in the *GLUD1* gene are the second most common cause of hyperinsulinemic hypoglycemia during infancy [3, 4] with an estimate of 1 in 200,000 (ORPHA, 35878). This rare genetic disease gives rise to the hyperinsulinism-hyperammonemia (HI/HA) syndrome that is caused by activating mutations in the *GLUD1* gene. This gene, located on chromosome 10q23.3, is composed of 13 exons and encodes the mitochondrial enzyme glutamate dehydrogenase (GDH). GDH catalyzes the reversible reaction α-ketoglutarate + NH_3_ + NADH ↔ glutamate + NAD^+^, the predominant flux of the reaction being tissue dependent [5]. When fueling the tricarboxylic acid cycle in the anaplerotic direction, the reaction serves as energy supplier by means of glutaminolysis. GDH is allosterically regulated, in particular by the inhibitory action of GTP and the activator ADP [6].

The clinical importance of the complex GDH allosteric regulations was originally uncovered by the discovery of a severe hypoglycemic disorder in children [7]. The phenotype of the patients is heterogeneous with neonatal and early infancy-onset of hypoglycemia, as well as elevations of plasma ammonia concentrations. *GLUD1* heterozygous pathogenic variants are mainly located in the region of the GTP-binding domain or in antenna-related region. Those gain-of-function variants produce an increase in GDH activity through reduced GTP-mediated inhibition of the enzyme [8] or higher sensitivity to the allosteric activator ADP [9]. Some of these activating variants of GDH are associated with both HI/HA and epilepsy [10]. Patients suffering from HI/HA syndrome display protein-induced insulin secretion, fasting hypoglycemia and increased ammonia levels independent of protein consumption. About 70% of patients are carriers of a de novo mutation; 30% are familial cases with autosomal dominant inheritance. Our study aims at the functional and enzymatic characterization of the GDH-G446V variant (alias c.1496G>T, p.(Gly499Val) (NM_005271.4)) in lymphoblastoid cells derived from a patient identified with this pathogenic point variant.

## Materials and methods

### Genetic analysis

Exome sequencing at the Genome Clinic of the University Hospitals of Geneva was performed as previously described (PMID, 25691535). Targeted bioinformatics analysis of a panel of 10 genes involved in congenital hyperinsulinism (*ABCC8*, *KCNJ11*, *GLUD1*, *GCK*, *HADH*, *HNF4A*, *HNF1A*, *SLC16A1*, *UCP2*, *CDKN1C*) was done through locally developed pipelines, which select only the variants from the genes of interest, masking the rest of the data. Variants filtering and classification was performed based on the guidelines for the interpretation of sequence variants from the American College of Medical Genetics and Genomics and the Association for Molecular Pathology (PMID, 25741868).

### Patient-derived cell preparation

Lymphocytes were isolated from peripheral blood of a HI/HA patient carrying the GDH-G446V variant and of a control subject (healthy non-carrier parent) with wild-type GDH. Lymphocytes were also isolated from three additional subjects for the assessment of variability of GDH activity among various individuals. Lymphoblastoid cell lines (LCL) were established by in vitro transduction of lymphocytes B with Epstein–Barr virus [11, 12] and cultured in RPMI-1640 medium at 11.1 mM glucose supplemented 10% (vol/vol) heat-inactivated fetal calf serum (FCS), 100 U/ml penicillin, 100 μg/ml streptomycin medium.

### GDH activity assay

LCLs were washed with PBS and resuspended in lysis buffer containing 50 mM Tris/HCl, 2 mM CDTA, and 0.2% Tween20 (pH 9.5). The cells were lysed by sonication. The GDH activity in lymphoblast homogenates was determined by the NADH autofluorescence using a Fluostar Optima (BMG Labtech) in 50 mM Tris/HCl buffer (pH 9.5) containing 2.6 mM EDTA and 1.4 mM NAD^+^ [13]. The reaction was initiated by the addition of 5 mM glutamate and effects of allosteric regulators GTP (1–100 μM) and ADP (300–1200 μM) were tested. The concentrations of the reactants and effectors were based on the estimated mitochondrial matrix volume of about 1 μL/mg protein, with NAD(H) in the range of 0.5–2.0 mM [14]. Protein concentrations were determined by Bradford assay.

### Metabolic function

Glycolytic and mitochondrial metabolic rates of control and mutant LCLs were assessed by the measurements of extracellular acidification rate (ECAR) and oxygen consumption rates (OCR), respectively, using the Seahorse XF^e^96 analyzer and the Mito stress kit (Agilent Technologies, Santa Clara, CA). After seeding in 96-well plates (8 × 10^3^ cells/well), cells were starved for 5.5 h in glucose-free and bicarbonate-free KRBH (pH 7.4) at 37 °C without CO_2_. They were then metabolically stimulated with GDH-dependent substrates 5 mM alanine and 5 mM glutamine for 10 min before blockade of mitochondrial respiration with 1 μM oligomycin, induction of maximal uncoupled respiration with 1 μM FCCP and finally inhibition of the electron transport chain (ETC) with 0.5 μM of both rotenone and antimycin A. The OCR and ECAR measurements are reported as pmoles/min and mpH/min, respectively.

### Modeling and energy state of the enzyme

Potential energy of wild type and mutant GDH was calculated using the CHARM22 algorithm [15]. The molecular models for GDH were constructed from human X-ray structure (PDB ID: 1L1F) superimposed to bovine X-ray crystal structures for open and closed states (PDB IDs: 3DJ2 and 3DJ4).

### Statistical analysis

Unless otherwise indicated, data are the means ± SD. Differences between groups were assessed by Student’s *t* test for single comparison and by one-way ANOVA for multiple comparisons (GraphPad, Prism 7.02). A *P* value lower than 0.05 was considered as significant.

## Results

### Clinical and genetic data

The baby girl, carrier of the GDH-G446V variant (OMIM # 606762), was born at term to non-consanguineous healthy parents of Eritrean origin after an uneventful pregnancy with a birth weight of 3.03 kg (P50), birth length 52 cm (P90), and head circumference of 34.5 cm (P50–P90). The patient has no siblings; there are no other diseases reported in the family. At the age of 4 months she was hospitalized because of hypoglycemic seizures, glucose levels were at 1.3 mM with concomitant insulin levels of 26.68 μIU/ml (= 185.3 pM) compatible with congenital hyperinsulinism. Pancreatic ultrasound showed a normal-sized pancreas. Treatment with diazoxide was started at a dose of 15 mg/kg per day. The patient responded to diazoxide and the treatment was continued throughout childhood and adolescence at 50 mg tid, since a decrease to 50 mg bid lead to hypoglycemia of 1 mM at the age of 10 years. Additional multiple hypoglycemic seizures with loss of consciousness were observed during young adulthood. Mild intellectual deficiency was attributed to the multiple hypoglycemic episodes. At the age of 26 years, the patient got pregnant while treated with diazoxide. At 5 weeks of gestation, she was briefly hospitalized, the blood results showed a random plasma glucose level of 3.4 mM, HbA1c of 25 mmol/mol (4.4%), and an ammonium level at 118 μM (reference range 11–35). Blood pressure was 105/69 mmHg. A treatment change to octreotide was discussed because of the described embryotoxicity of diazoxide, classified as pregnancy category C according to the FDA, but the patient refused this proposal. At 11 weeks of pregnancy a trial of diazoxide discontinuation was started in association with a continuous glucose monitoring system (Dexcom G4®, Dexcom, Inc., San Diego, CA, USA) in order to be alerted in case of hypoglycemia. The system recorded less than 48 h before the occurrence of a technical problem. Four percent of the values were below 4.4 mM, but no hypoglycemia was noted according to the definition of the international hypoglycemia study group [16]. Two days after treatment suspension, a severe hypoglycemic episode (glucose of 1.3 mM) occurred with loss of consciousness and treatment was reintroduced.

Exome analysis revealed a de novo heterozygous pathogenic variant in the *GLUD1* gene: c.1496G>T, p.Gly499Val (NM_005271.4), referred to as GDH-G446V (see Supplemental Figure.S1). No other pathogenic or likely pathogenic variant in the gene panel analyzed was identified in the index case. Confirmation and segregation analysis of the *GLUD1* variant were performed by Sanger sequencing (in the patient, her parents, and her own daughter).

Patient’s daughter was born at term with intra-uterine growth restriction (IUGR) with a birth weight of 2.41 kg (< 2SD), a birth length of 46 cm (< 2SD) and a head circumference of 32.5 cm (< 2SD). Cord blood glucose was 4.0 mM with a C-peptide level of 382 pM (reference range 370–1470); ammonium level was 110 μM (reference range 0–140). Genetic analysis revealed that the daughter did not carry the *GLUD1* pathogenic variant. Accordingly, the IUGR could be due to either limited metabolic fuel in the context of maternal hypoglycemia or, since diazoxide passes the placental barrier, a direct effect on fetal growth or an indirect effect via its hypotensive action potentially leading to placental hypoperfusion. The child’s developmental outcome will be followed closely.

### Mitochondrial metabolism

**Fig. 1:**
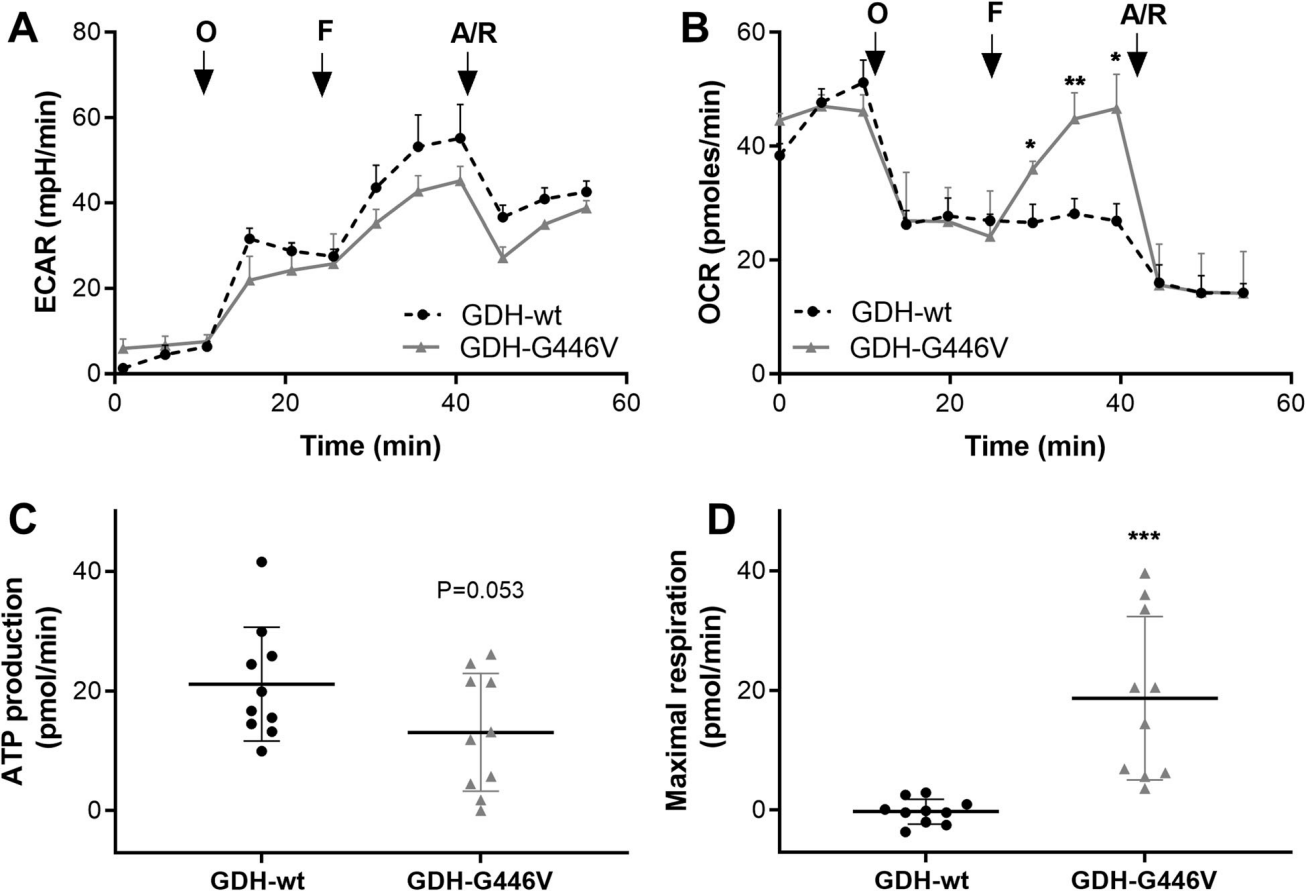
Mitochondrial metabolism in EBV-transformed lymphoblasts. The metabolic function of LCL lymphoblasts derived from control subject (GDH-wt) or from patient with mutant GDH (GDH-G446V) assessed using a 96-well plate Seahorse analyzer. Extracellular acidification rate (ECAR, **a**) and the oxygen consumption rate (OCR, **b**) were measured under the stimulation with 5 mM alanine and 5 mM glutamine, followed by the sequential addition of oligomycin (O), FCCP (F), and antimycin A/rotenone (A/R). ATP production (**c**) and maximal respiration (**d**) were calculated from OCR raw data. Values are shown as means ± SD, *n* = 10, *P < 0.05, **P < 0.01, ***P < 0.001 for wild-type GDH-wt versus GDH-G446V

In order to study the effects of the GDH-G446V variant on cell metabolism, patient-derived LCLs were established from lymphocytes isolated from the HI/HA patient and from control subject (healthy non-carrier parent). Their respective metabolic profiles were assessed by simultaneous measurements of the extracellular acidification rate (ECAR, an indicator of glycolytic rate) and the oxygen consumption rate (OCR, an indicator of mitochondrial oxidative phosphorylation). Stimulated with GDH-dependent substrates (glutamine and alanine), both GDH-wt and GDH-G446V LCLs relied mostly on mitochondrial respiration rather than anaerobic glycolysis for energy provision (Fig. 1a, b). Inhibition of the mitochondrial ATP synthase (complex V) by oligomycin switched the source of energy provision from mitochondrial oxidative phosphorylation toward the induction of glycolysis and lactate production, as shown by the drop in the OCR and increase in ECAR, respectively (Fig. 1a, b). The change was similar in GDH-wt and GDH-G446V LCLs. However, induction of maximal respiration by a mitochondrial uncoupler prompted robust OCR in mutant cells as opposed to control LCLs. The associated further increase in ECAR was less pronounced in GDH-G446V versus GDH-wt cells. Finally, inhibition of complexes I and III confirmed that the elevated respiration in mutant cells was indeed contributed by mitochondria. These data show that the overall ATP production was not affected by GDH-G446V (Fig. 1c), while the maximal mitochondrial respiration was enhanced (Fig. 1d). Therefore, the G446V mutation increased the capacity of mitochondrial metabolism in response to GDH-dependent substrates.

### Energetics of wtGDH and G446VGDH

To get insight into molecular dynamics of hyperactivating mutation, the difference in potential energy was calculated between opened and closed structures for GDH-G446V and GDH-wt. CHARM22 algorithm was used for calculations [15]. The energy barrier between opened and closed state in GDH-G446V was 59% of the GDH-wt (Supplemental Table). This might favor substrate/product turnover in the mutant enzyme, which is indicative of a hyperactivity phenotype. The molecular models for GDH were derived from X-ray crystal structure for opened and closed state (PDB IDs: 3DJ2 and 3DJ3).

**Fig. 2:**
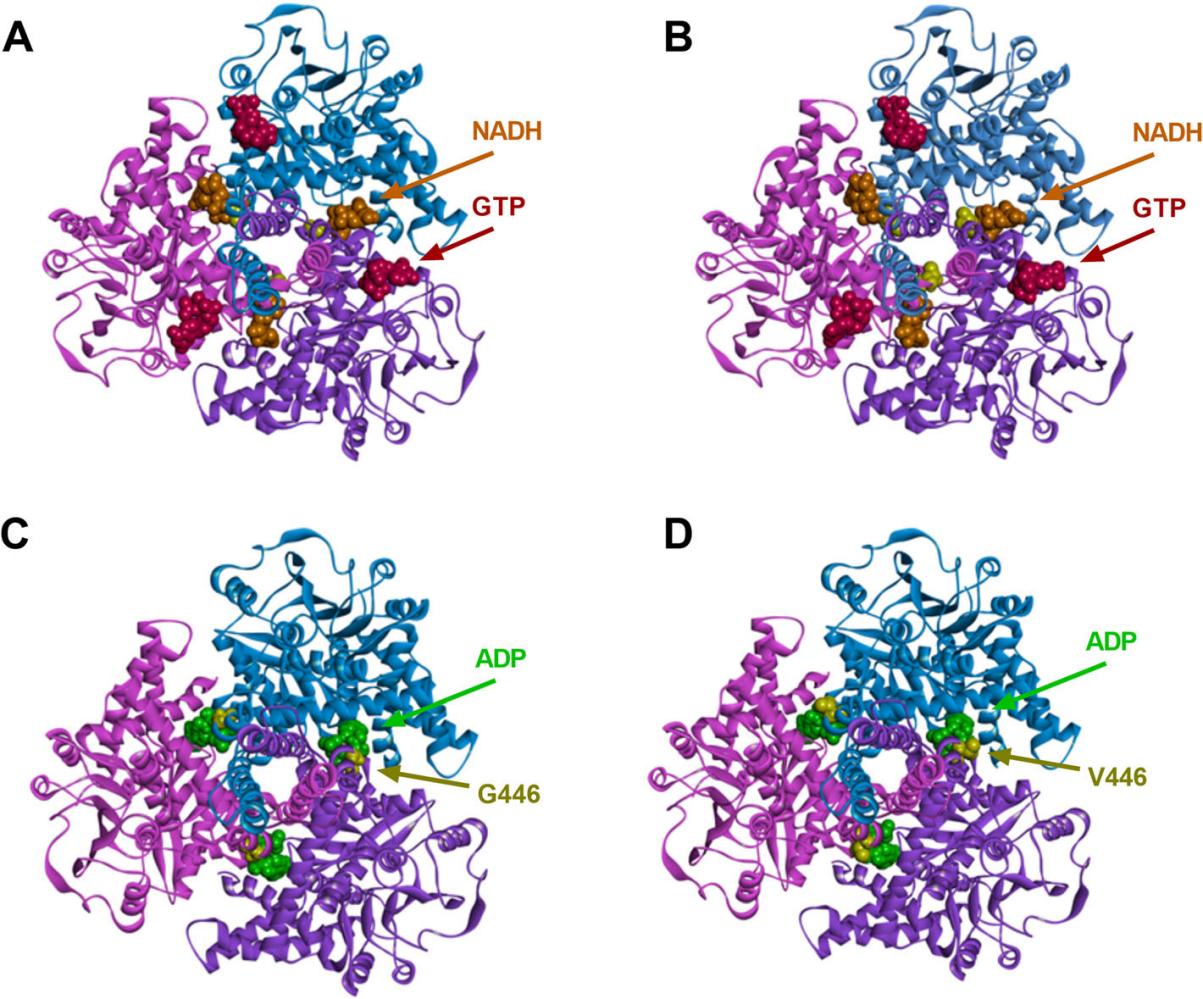
GDH wild type and p.G446V molecular structures. Molecular models for closed (**a**, **b**) and opened (**c**, **d**) conformations of GDH wild type (left) and mutated form p.G446V (right) based on the structure of human GDH (PDB: 1L1F) aligned to open and closed structures of bovine GDH derived from cryo-electron microscopy [19] (PDB: 3DJ2 and 3DJ4). Different colors highlight each monomer of the trimer of GDH assembly displayed from the top view with location of the mutation p.G446V (yellow ball structure) and co-factors (balls structures: orange NADH and red GTP). (**c**, **d**) Single subunit of GDH (side view) with highlighted mutation on the descending helix

GDH is a homohexamer, structurally composed of two trimers here depicted as schematic ribbon diagram (Fig. 2, top view of the trimer, GDH-wt and GDH-G446V). GTP (location, see Fig. 2a, b) allosterically inhibits the enzyme, while ADP (location, see Fig. 2c, d) is an activator. Computational simulation of GDH-G446V model indicated conformational changes of both opened (ADP binding) and closed states (GTP binding) versus GDH-wt, pointing to altered allosteric regulation in the mutant enzyme.

### Enzymatic properties of GDH-G446V mutant

**Fig. 3:**
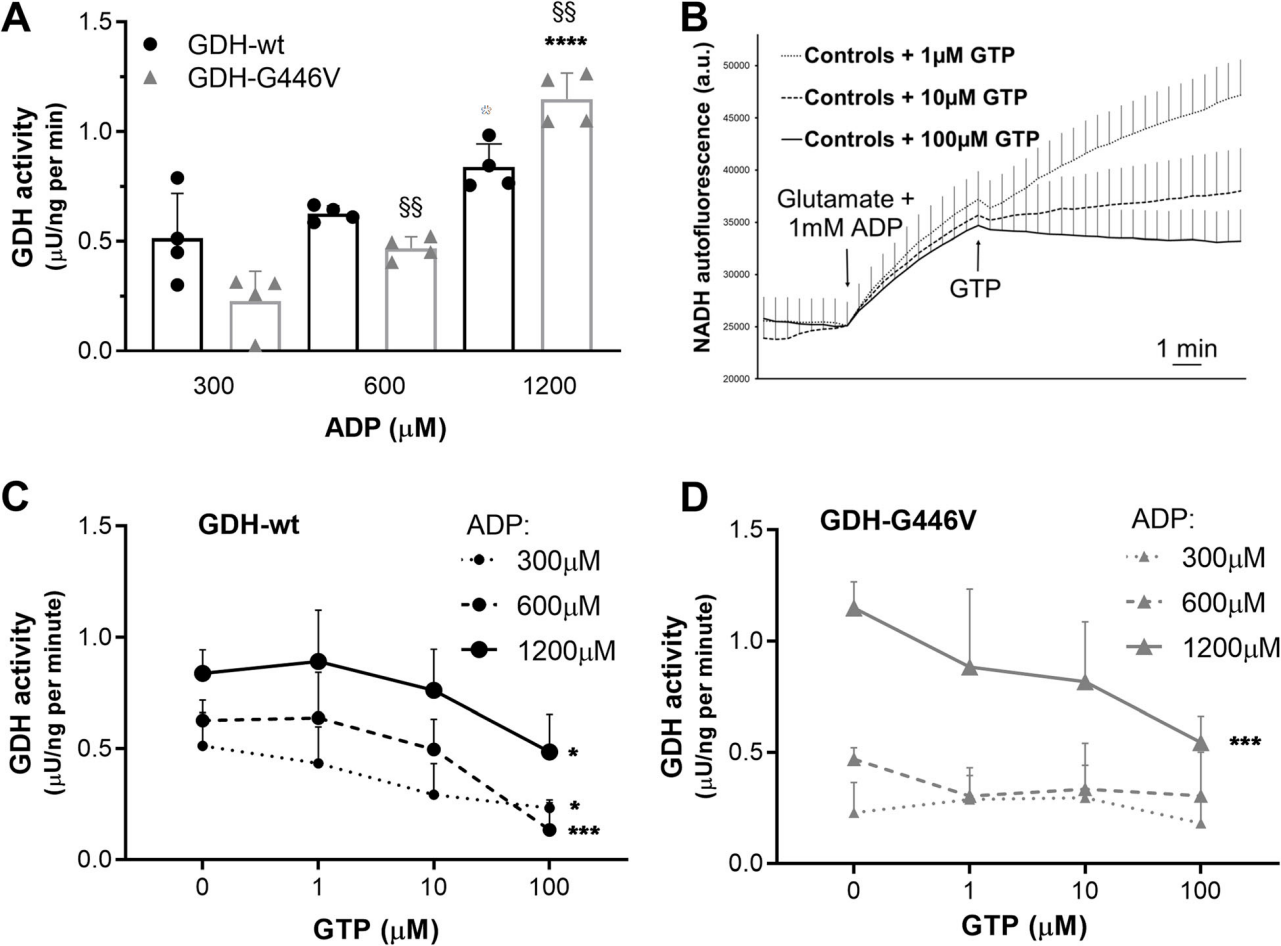
Enzymatic properties of GDH-G446V mutant. Effects of allosteric modulators on GDH activities in EBV-transformed lymphoblasts from control subjects with GDH-wt and patient with mutant GDH-G446V. Enzymatic assays were performed on lymphoblast homogenates using 5 mM glutamate and ADP (300–1200 μM) as allosteric activator in the absence of GTP (**a**). Sensitivity to different concentrations of GTP (1–100 μM) tested on 4 control subjects (2 males, 2 females) of different ages (28–52 years old) at 5 mM glutamate plus 1000 μM ADP (**b**). GTP dose responses conducted at different concentrations of ADP on GDH-wt cells (**c**) and mutant GDH-G446V cells (**d**). Values are means ± SD, *n* = 4; **a**, *P < 0.05 and ****P < 0.0001 versus 300 μM ADP condition of corresponding genotype, ^§§^*P* < 0.001 versus GDH-wt of corresponding assay condition; **c**, **d** *P < 0.05 and ***P < 0.001 versus 0 μM GTP condition

The enzymatic activities of GDH-wt and GDH-G446V were measured in cell homogenates of lymphoblasts derived from control subjects and HI/HA patient, respectively. In particular, we assessed the sensitivity to allosteric regulators, i.e., the inhibitory effect of GTP and the activation by ADP. Both GDH-wt and mutant GDH-G446V were responsive to a dose response of ADP (Fig. 3a). However, in the presence of 1200 μM ADP, GDH-G446V exhibited higher activity compared to GDH-wt. In control GDH-wt cells, a dose response of GTP showed the strong inhibitory action of this nucleotide at 100 μM (Fig. 3b). Different control subjects (males and females of different ages) exhibited similar responses to the inhibitory action of GTP (see Supplemental Figure S2). This GTP effect was effective at all of the tested concentrations of the activator ADP (Fig. 3c). In contrast, as shown in Fig. 3d, GDH-G446V cells were responsive to the inhibitory action of 100 μM GTP only in the presence of the highest concentration of ADP (1200 μM).

## Discussion

The enzymatic characterization revealed that mutant GDH-G446V exhibits reduced sensitivity to both ADP and GTP in the higher range of their respective mitochondrial concentrations. This indicates that the mutant GDH would be more active in energized mitochondria (high GTP, low ADP), as well as in de-energized mitochondria (low GTP, high ADP). Such a pattern points to tissue-specific responses of mutant GDH-G446V, with a neutral inflection point at intermediate concentrations of the allosteric regulators.

**Fig. 4:**
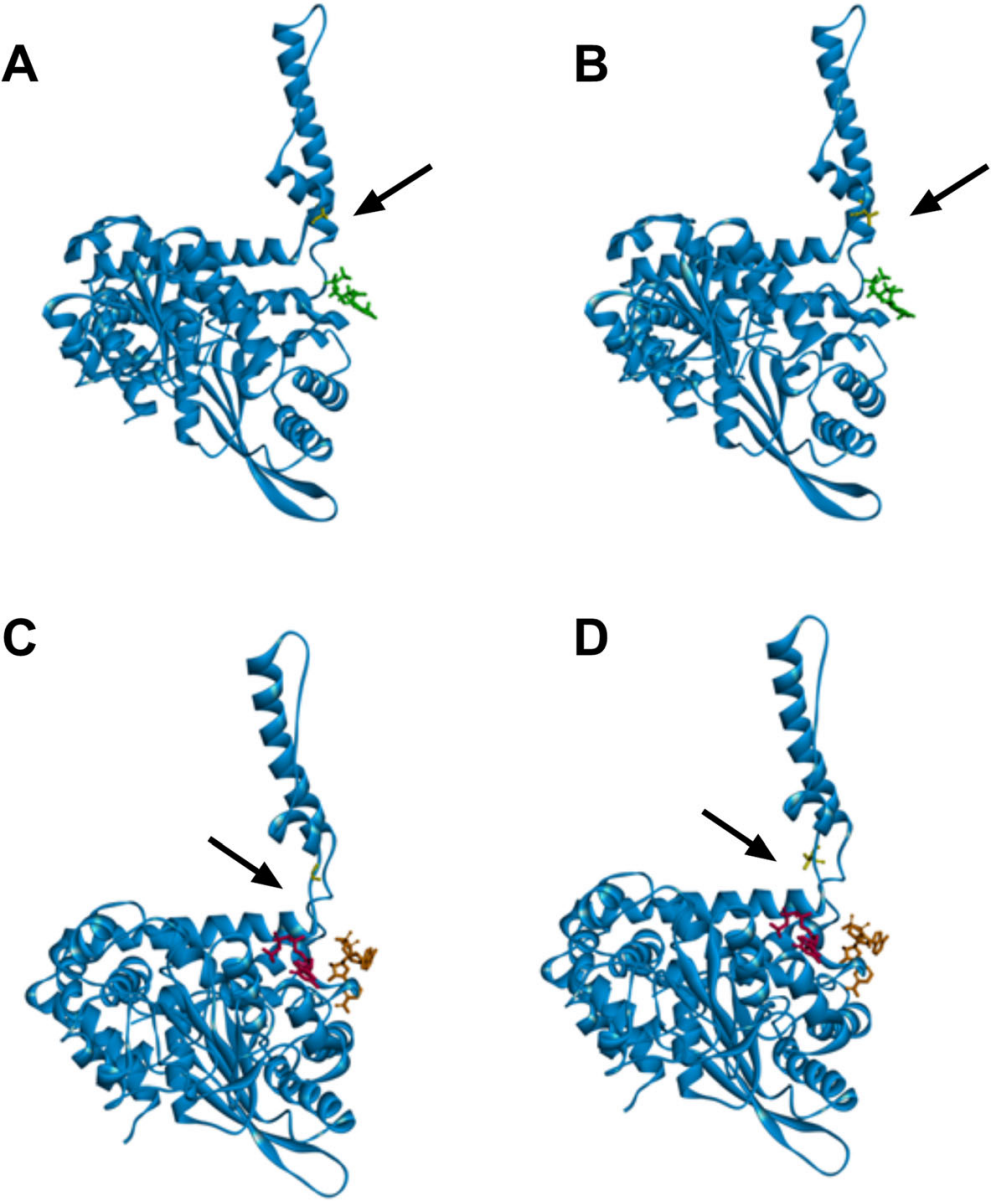
Molecular structures of GDH-wt and GDH-G446V subunit. Single subunit of GDH (side view, blue) with highlighted glycine-446 (yellow) in wtGDH (arrow **a**, **b**) and 446-valine (yellow) in GDH-G446V (arrow **c**, **d**) on the descending helix in opened (**a**, **c**) and closed (**b**, **d**) conformation. The pivot helix of the GDH-G446V shows a different conformation in the closed state (**b**, **d**) versus the GDH-wt

The allosteric regulation of GDH is facilitated by subunit interactions contributed mainly by the antenna region, which ascends via helix structure and descends through random coil structure. Helices of the trimer wrap around each other permitting high degree of intercalation between them, thereby playing a key role in the opened/closed conformational changes of the enzyme. Glycine to valine 446 mutation is located on the pivot of the helix, i.e., at the basis of the antenna (Fig. 4). Glycine is the amino acid with the smallest side chain and its presence introduces molecular flexibility to this region of GDH (Fig. 4a, b). On the contrary, valine is a hydrophobic branched-chain amino acid that can cause a steric interference upon clockwise motion of intercalated domains during turnover of GDH (Fig. 4c, d). Of note, the GDH-G446V variant confers a different conformation of the pivot helix in the closed state (Fig. 4b, d) versus the GDH-wt, probably due to compromised rotation. Most of the HI/HA patients present sporadic pathogenic variants and are therefore heterozygous. Accordingly, the respective contributions of wild type versus mutant subunits in the operating GDH hexamer are unknown. One can hypothesize that GDH-G446V patients carry heterohexamers composed of both wild type and mutant mers. Because of the expression of a strong phenotype, the mutant form appears to be dominant over wild-type subunits.

## Conclusions

Authors have previously reported substitutions of glycine at position 446 for serine and aspartate [7], for arginine and valine [8], and for cysteine [17]. All of these pathogenic variants are associated with HI/HA, giving rise to impaired GTP-mediated inhibition of GDH enzymatic activity. The G446V was not yet characterized at the molecular and cellular levels. Our results show that the glycine to valine substitution at position 446 altered the GDH allosteric sensitivity to both inhibitory action of GTP and activation by ADP. In terms of cellular energetics, this was translated into increased mitochondrial metabolism in the presence of amino acids. Such an effect may render the pancreatic ß-cell responsive to glutamine [18], leading to inappropriate insulin secretion when the blood glucose is not stimulatory. The stimulation of insulin secretion in such a situation induces deleterious hypoglycemia. In hepatocytes, gain-of-function variant of GDH promotes higher ammonia production, contributing to the hyperammonemia [9]. Pathogenic variants in the *GLUD1* gene illustrate the complexity of allosteric regulations and the tissue specificities of the enzyme GDH, possibly contributed by the actual concentrations of the allosteric effectors, namely GTP and ADP. In situ investigations should be conducted to dig into these convoluted mechanisms.

## Supplementary information

**Additional file 1: Supplemental Table.** Potential energy calculated for opened and closed state of GDH-wtand GDH-G446V. **Supplemental Figure S1.** GLUD1genomic DNA sequence. Analysis shows c.1496G>T variant in the DNA of the HI/HA patient. **Supplemental Figure S2**. Effects of the allosteric modulator GTP on GDH activity in EBV-transformed lymphoblasts from 4 control subjects; 2 males in their 6th(M50+) and 4th(M30+) decade of age and 2 females in their 3rd(F20+) and 5th(F40+) decade. Enzymatic assays were performed on lymphoblast homogenates using 5mM glutamate and 1mM ADP as allosteric activator in the presence of 1-100 μM GTP.

## Data Availability

Data will be uploaded to a public repository after manuscript acceptance (https://yareta.unige.ch//frontend/).
