# Correction to: Hyperinsulinism associated with *GLUD1* mutation: allosteric regulation and functional characterization of p.G446V glutamate dehydrogenase

**DOI:** 10.1186/s40246-021-00305-8

**Published:** 2021-01-25

**Authors:** Karolina Luczkowska, Caroline Stekelenburg, Frédérique Sloan-Béna, Emmanuelle Ranza, Giacomo Gastaldi, Valérie Schwitzgebel, Pierre Maechler

**Affiliations:** 1 Department of Cell Physiology and Metabolism, University of Geneva Medical Center, 1206 Geneva, Switzerland; 2 Faculty Diabetes Center, University of Geneva Medical Center, 1206 Geneva, Switzerland; 3 Pediatric Endocrine and Diabetes Unit, Department of Pediatrics Gynecology and Obstetrics, University Hospitals of Geneva, Geneva, Switzerland; 4 Department of Genetic Medicine and Development, Faculty of Medicine, University of Geneva, 1211 Geneva, Switzerland; 5 Department of Genetic Medicine and Laboratory, University Hospitals of Geneva, 1211 Geneva, Switzerland; 6 Division of Endocrinology, Diabetology, Hypertension and Nutrition, Geneva University Hospitals, 1211 Geneva, Switzerland

**Correction to: Hum Genomics 14, 9 (2020)**

**https://doi.org/10.1186/s40246-020-00262-8**

Following publication of the original article [1], the authors would like to correct the spelling of the PDB entries when describing the X-ray crystal structures for open and closed states in the section *Modeling and energy state of the enzyme*, section *Energetics of wtGDH and G446VGDH* and *Fig. 2’s caption*.

Two letters of the PDB entries have been inverted. In the published version, it wrongly shows: 3DJ2 and 3DJ4. However, open and closed states should be 3JD2 and 3JD4.

The original article [1] has been corrected.
