# Supplementary material for: Hyperinsulinism associated with GLUD1 mutation: allosteric regulation and functional characterization of p.G446V glutamate dehydrogenase
Source: Hum Genomics. 2020 Mar 6;14:9. doi: 10.1186/s40246-020-00262-8 (PMC7060525; doi:10.1186/s40246-020-00262-8)

Supplemental Figure 1: ***GLUD1* genomic DNA sequence**. Analysis shows c.1496G>T variant in the DNA of the HI/HA patient

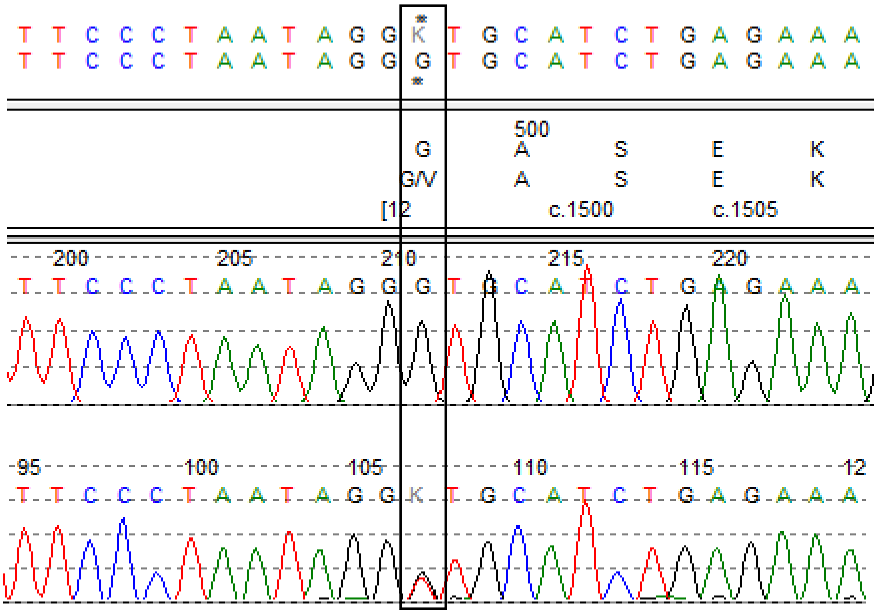

Reference

Patient

Supplemental Table: Potential energy calculated for opened and closed state of GDH-wt and GDH-G446V.

Genotype	Opened [kcal/mol]	Closed [kcal/mol]	Difference
GDH-wt	24’419	24’650	231
GDH-G446V	24’419	24’555	136 (59% of wt)

Supplemental Figure 2: Effects of the allosteric modulator GTP on GDH activity in EBV-transformed lymphoblasts from 4 control subjects; 2 males in their 6th (M50+) and 4th (M30+) decade of age and 2 females in their 3rd (F20+) and 5th (F40+) decade. Enzymatic assays were performed on lymphoblast homogenates using 5mM glutamate and 1mM ADP as allosteric activator in the presence of 1-100 µM GTP.

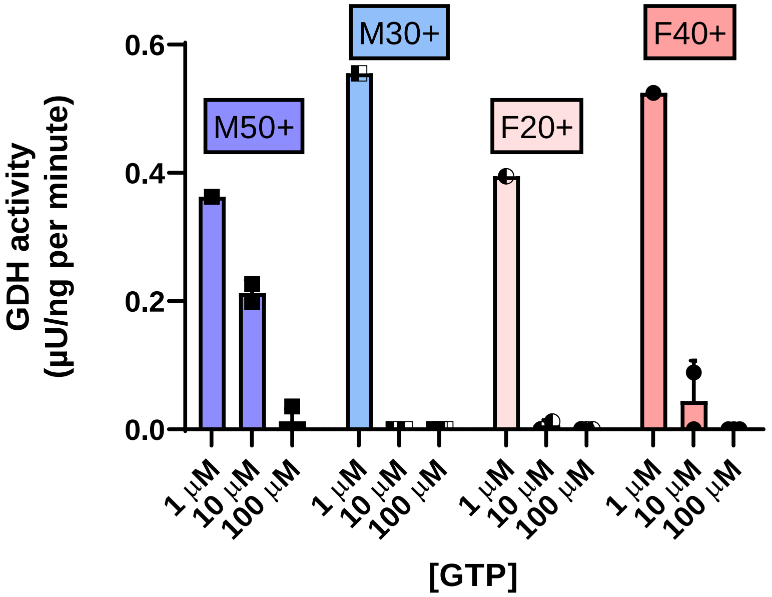

Supplement: Supplementary file 1 — Additional file 1: Supplemental Table. Potential energy calculated for opened and closed state of GDH-wtand GDH-G446V. Supplemental Figure S1. GLUD1genomic DNA sequence. Analysis shows c.1496G>T variant in the DNA of the HI/HA patient. Supplemental Figure S2. Effects of the allosteric modulator GTP on GDH activity in EBV-transformed lymphoblasts from 4 control subjects; 2 males in their 6th(M50+) and 4th(M30+) decade of age and 2 females in their 3rd(F20+) and 5th(F40+) decade. Enzymatic assays were performed on lymphoblast homogenates using 5mM glutamate and 1mM ADP as allosteric activator in the presence of 1-100 μM GTP. [file 40246_2020_262_MOESM1_ESM.pdf]
